# Supplementary material for: Serial dependence is absent at the time of perception but increases in visual working memory
Source: Sci Rep. 2017 Nov 7;7:14739. doi: 10.1038/s41598-017-15199-7 (PMC5677003; doi:10.1038/s41598-017-15199-7)
Supplement: Supplementary file 1 — Supplementary Information [file 41598_2017_15199_MOESM1_ESM.pdf]

Serial dependence is absent at the time of perception but increases in visual working memory: Supplementary information

Daniel P. Bliss[1,*], Jerome J. Sun[1], and Mark D'Esposito[1,2]

[1]UC Berkeley, Helen Wills Neuroscience Institute, Berkeley, CA, USA
[2]UC Berkeley, Department of Psychology, Berkeley, CA, USA
[*]Corresponding author: dbliss@berkeley.edu

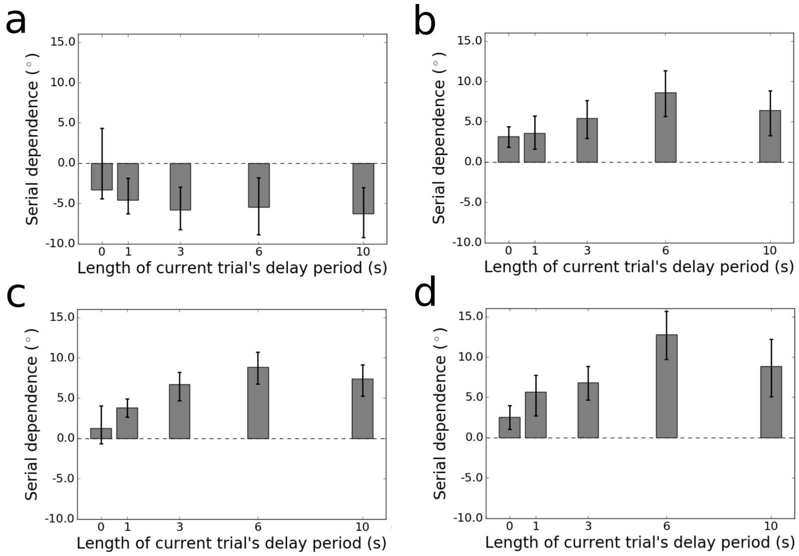

Supplementary Figure 1. (A-D) Magnitude of serial dependence in the data from four sample subjects for each delay period tested in Experiment 1. For all of these subjects, serial dependence was measured as the peak-to-peak of a least squares fit of the derivative of Gaussian (DoG) tuning function to the data. Error bars represent bootstrapped 95% confidence intervals. (A) Data from the same subject used for Figure 4B, who showed prominent adaptation (negative serial dependence) when all delay conditions were combined. Separating the delay conditions for this subject reveals no evidence of positive serial dependence, and an adaptation effect that persists at an approximately constant level throughout all delays tested. (B) Data from the same subject used for Figure 4D, who showed prominent positive serial dependence. The evolution serial dependence over the working memory delay in this subject reflects the group-level effect – a gradual increase in the strength of serial dependence that asymptotes around 6 s. (C-D) These panels depict data from two subjects whose serial dependence effect was strongly positive when delay conditions were collapsed (the two participants farthest to the right in Figure 4A, in order). As for the subject highlighted in panel B, the trajectory of the effect in these two subjects parallels that of the group data – a clear scaling with working memory delay length that asymptotes between 3 and 10 s. Together, these exemplar subjects represent the two extremes of the spectrum of trial-history effects (with unusually clean patterns of the effects over delay length). Though we hesitate to generalize from this small subset of our full dataset, one possibility that emerges from these plots is that group-level behavior reflects the summation of two processes happening simultaneously (with different relative strengths in different subjects): an adaptation effect that changes little as perception gives way to working memory, and a positive serial dependence effect that comes online and gets stronger as working memory is engaged. These plots suggest, furthermore, that positive serial dependence may occur for some subjects as early as the time of perception (panels B and D), if adaptation is especially weak. That said, early engagement of the working memory system – rather than purely perceptual processes – may drive this effect, when it is present.

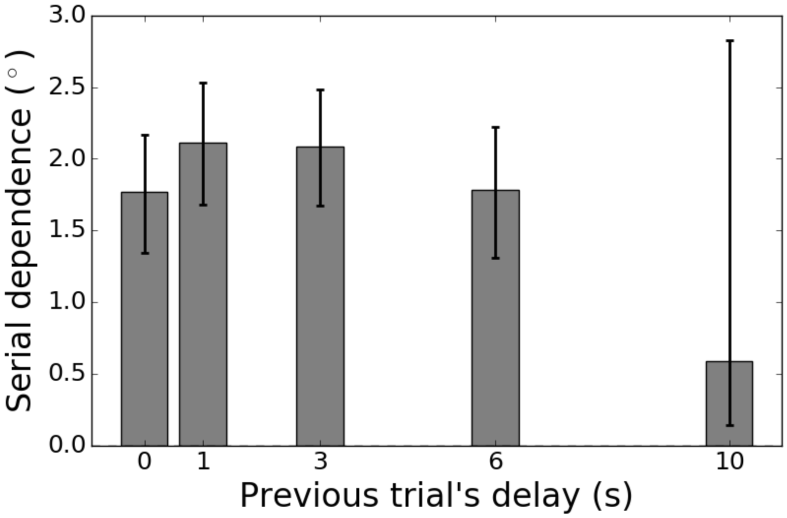

Supplementary Figure 2. Peak-to-peak of the DoG fit to the group data for each length of the previous trial's delay tested in Experiment 1. Error bars represent bootstrapped 95% confidence intervals. Note that this plot qualitatively replicates Figure 5A – which displays the Clifford model fits for the same data – but reveals the instability of resampling statistics that use the DoG fit for the 10-s condition (where the peaks of the "peripheral bumps" in the tuning of serial dependence are larger than those of the central attractive bias; see Fig. 5C). The estimated confidence interval is much wider than the estimated magnitude of the effect.

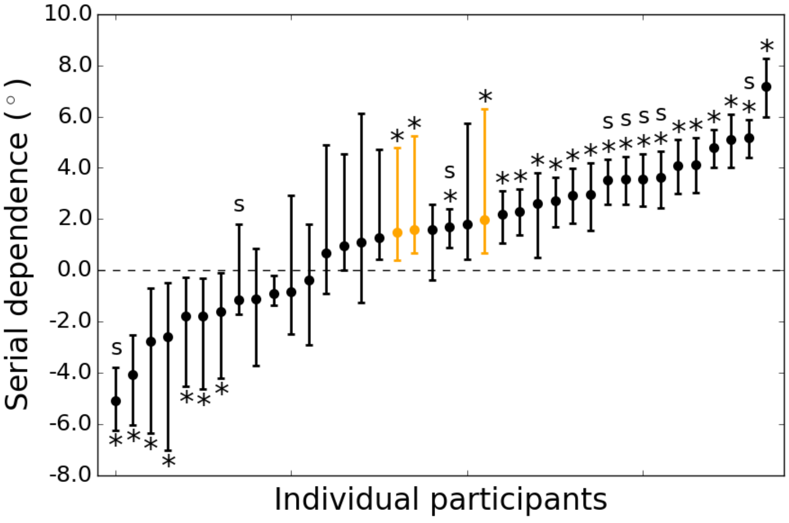

Supplementary Figure 3. Magnitudes of serial dependence observed for the individual participants tested in Experiment 1, with monitor size labeled. This is a reproduction of Fig. 4A, but with an additional symbol (s) that labels subjects who completed the experiment using a 17-in computer monitor rather than the 23-in monitor used for the remaining subjects. Subjects that used the smaller monitor span the range of serial dependence effects, with one showing prominent adaptation, one showing an absence of consistent trial-history effects, and the remainder scattered throughout the range from weak to strong positive serial dependence. The mean peak-to-peak of serial dependence in the group that used the 17-in monitor was $1.9^\circ \pm 1.1^\circ$ SEM, compared to $1.2^\circ \pm 0.5^\circ$ SEM for the 23-in monitor.
